# The complete mitochondrial genome of *Aclees taiwanensis* Kôno, 1933 (Coleoptera: Curculionidae)

**DOI:** 10.1080/23802359.2022.2107440

**Published:** 2022-08-10

**Authors:** Ki-Jeong Hong, Woong Ki, In-Jun Lee, Hyobin Lee, Jongsun Park, Wonhoon Lee

**Affiliations:** a Department of Plant Medicine, Sunchon National University, Suncheon, Korea; b Department of Plant Medicine, Gyeongsang National University, Jinju, Republic of Korea; c Infoboss Inc, Seoul, Republic of Korea; d Infoboss Research Center, Seoul, Republic of Korea; e Institute of Agriculture and Life Science, Gyeongsang National University, Jinju, Republic of Korea

**Keywords:** Mitochondrial genome, *Aclees taiwanensis*, Curculionidae, Coleoptera, Korea

## Abstract

We sequenced the complete mitochondrial genome of *Aclees taiwanensis* collected in Korea. The circular mitogenome of *A. taiwanensis* is 17,435 bp, longer than that of *Aclees cribratus*, and includes 13 protein-coding genes, two ribosomal RNA genes, 22 transfer RNAs, and a control region/D-loop. The AT ratio is 75.4%. Maximum-likelihood and Bayesian inference phylogenetic trees showed that *A. taiwanensis* was clustered with *A. cribratus* with full-support values for both trees.

*Aclees taiwanensis* Kôno, 1933 (Coleoptera: Curculionidae), which is native to Asia (Meregalli et al. 2020), is an insect pest of *Ficus* spp.: it is especially a major threat for the fig tree, *Ficus carica* (Farina et al. 2020). In Korea, this species was firstly reported on *F. carica* in the Haenam-gun district of Jeonnam Province in 2020, and is considered one of the most invasive species recently introduced to Korea (Hong et al. 2020). To study genetic variations with *A. taiwanensis* samples from other countries, we sequenced the first complete mitogenome of *A. taiwanensis* collected in Korea, which can be used to track their origin in the integrated systems such as the Integrated Platform for Invasive Pests (Park, Kim, Xi, et al. 2020).

We extracted genomic DNA from one *A. taiwanensis* individual (34°40′95.80ʺN, 126°53′17.80ʺE; Songji-myeon, Haenam-gun, Jeollanam-do, Republic of Korea) using a DNeasy Blood & Tissue Kit (QIAGEN, Hilden, Germany). The voucher specimen was deposited at the Sunchon National University (2020HKJ#1001, Contact: Dr. Ki-Jeong Hong, curcul@scnu.ac.kr). The ethical approval or other relevant permission is not need in this study because *Aclees taiwanensis* is a common species in Korea and other countries. In total, 5.8 Gbp of raw sequence data was obtained using an Illumina NovaSeq6000 (Macrogen Inc., South Korea) by constructing a pair-end library of 350 bp inserted size (151 bp reads) that were filtered by Trimmomatic v0.33 (Bolger et al. 2014), and *de novo* assembled using Velvet v1.2.10 (Zerbino and Birney 2008). Gaps were closed with GapCloser v1.12 (Zhao et al. 2011), BWA v0.7.17, and SAMtools v1.9 (Li et al. 2009; Li 2013) available in the Genome Information System (http://geis.infoboss.co.kr/) utilized in the previous organelle genomic studies (Joo et al. 2020; Lee et al. 2020; Park, Xi, Oh, 2020; Jung et al. 2021; Park, Lee, et al. 2021). Coverage of raw data against the assembled mitochondrial genome was 571.34×. Raw reads used for assembling the mitochondrial genome were deposited into the NCBI SRA (SRA accession: SRR14621276). Geneious Prime® v2020.2.4 (Biomatters Ltd, Auckland, New Zealand) was used to annotate the mitogenome based on the *Aclees cribratus* Gyllenhal, 1835 mitogenome (GenBank accession: MT501538; Wang et al. 2020).

The *Acelees taiwanensis* mitogenome (GenBank accession: MZ305480) is 17,435 bp long, which is the longer than that of *A. cribratus* (Wang et al. 2020). It contains 13 protein-coding genes, two rRNAs, 22 tRNAs, and a control region/D-loop. Its nucleotide composition is AT-biased (A + T is 75.4%), similar to that of *A. cribratus* (75.8%; Wang et al. 2020). The gene order of *A. taiwanensis* is conserved in 27 Curculionidae mitogenomes.

Simple sequence repeats (SSRs), which have been utilized for distinguishing species (Simon et al. 1994; Mousson et al. 2005) or for identifying cryptic species (Burger et al. 2014), were investigated on the *A. taiwanensis* mitogenome using the SSRDB (http://ssrdb.infoboss.co.kr/) used in previous studies (Kim et al. 2019; Lee et al. 2020; Park, Xi, and Kim, 2020; Park, Xi, Kim, et al. 2020; Choi et al. 2021; Kim et al. 2021). In total, 17 normal SSRs (18.89%), 65 potential SSRs (72.22%), and eight extended SSRs (8.89%) were identified, which represent a similar distribution to that of *Figulus binodulus* Waterhouse, 1872 (Coleoptera: Lucanidae) (Lee et al. 2020). Four monoSSRs and six diSSRs were identified as suitable first targets to develop molecular markers for distinguishing between *A. taiwanensis* populations because monoSSRs and diSSRs usually displayed the variations in the number of repeats within species, such as various insect species including *Sogatella furcifera* (Horva'th) (Hemiptera: Delphacidae)(Park, Min, Kim, et al. 2021), and *Stegobium paniceum* (Linnaeus, 1761) (Coleoptera: Ptinidae; Anobiinae) (Park et al. 2022).

We inferred the phylogenetic relationship of 27 Curculionidae mitogenomes for which annotation was available and species had been identified, including the *A. taiwanensis* mitogenome and one outgroup species, *Cyllorhynchites ursulus* (Roelofs, 1874) (Coleoptera: Rhynchitinae) (Kim and Lee 2018). Multiple sequence alignments of each conserved PCGs were conducted using MAFFT v7.450 (Katoh and Standley 2013) and were merged for constructing phylogenetic trees. Bootstrapped maximum-likelihood (ML) with 1,000 pseudoreplicates and Bayesian Inference (BI) trees were constructed using MEGA X (Kumar et al. 2018) and MrBayes v3.2.6 (Ronquist et al. 2012), respectively, based on the concatenated multiple sequence alignment of the conserved genes. A heuristic search was adopted with the option of nearest-neighbor interchange branch swapping, the Tamura-Nei model, and uniform rates among sites for the ML tree with default values for the remaining options. The BI tree was constructed using the GTR model with gamma rates as the molecular model, Markov-chain Monte Carlo algorithm for 1,000,000 generations, and sampling trees every 200 generations, with four chains running simultaneously. Phylogenetic trees showed that *A. taiwanensis* was clustered with *A. cribratus* with full supportive values of the ML and BI trees (Figure 1). In addition, both trees displayed that two subfamilies, Cryptorhynchinae and Molytinae, that were not monophyletic (Figure 1), suggesting that additional mitogenomes of Cryptorhynchinae and Molytinae subfamilies are required to confirm whether the two subfamilies are also paraphyletic or not.

**Figure 1.**
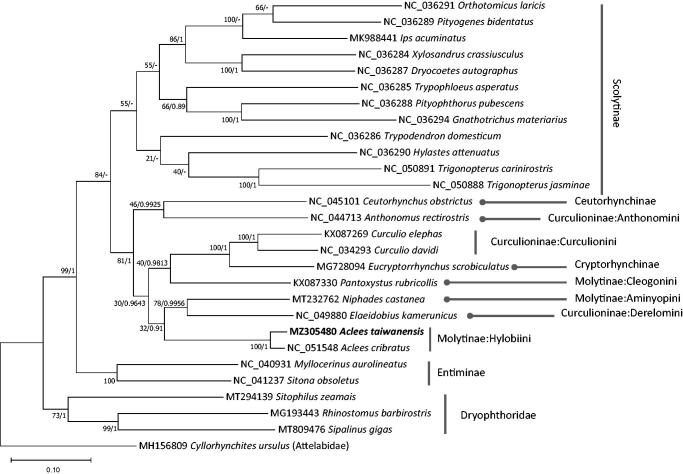
Maximum-likelihood (1,000 bootstrap repeats) and Bayesian Inference phylogenetic trees of 27 mitochondrial genomes of Curculionidae and one outgroup species. A phylogenetic tree was drawn based on the maximum-likelihood tree. Subfamily names are displayed with indicate lines in the right side of the tree and family name of the outgroup was displayed inside the bracket. The numbers above branches indicate supportive values of Maximum-likelihood and Bayesian Inference phylogenetic trees, respectively.

## Author contribution

The article was designed and conceived by Ki-Jeong Hong, Jongsun Park and Wonhoon Lee; Chao Woong Ki, In-Jun Lee collected and identified the insect material; Ki-Jeong Hong and Hyobin Lee contributed significantly to phylogenetic analysis and manuscript preparation; Jongsun Park and Wonhoon Lee were involved in the interpretation of the data and revised the manuscript critically for intellectual content. All authors approved the final version to be published and agreed to be accountable for all aspects of the work.

## Data Availability

Mitochondrial genome sequence can be accessed via accession number MZ305480 in GenBank of NCBI at https://www.ncbi.nlm.nih.gov. The associated BioProject, SRA, and Bio-Sample numbers are PRJNA731980, SRR14621276, and SAMN19304194, respectively.
